# Crystal structure and Hirshfeld surface analysis of 4-bromo-2-[3-methyl-5-(2,4,6-tri­methyl­benz­yl)oxazolidin-2-yl]phenol

**DOI:** 10.1107/S2056989022005928

**Published:** 2022-06-10

**Authors:** Ali N. Khalilov, Victor N. Khrustalev, Elena A. Fortalnova, Mehmet Akkurt, Sema Öztürk Yıldırım, Ajaya Bhattarai, İbrahim G. Mamedov

**Affiliations:** a "Composite Materials" Scientific Research Center, Azerbaijan State Economic University (UNEC), H. Aliyev str. 135, Az 1063, Baku, Azerbaijan; b Department of Chemistry, Baku State University, Z. Khalilov str. 23, Az, 1148 Baku, Azerbaijan; c Peoples’ Friendship University of Russia (RUDN University), Miklukho-Maklay St. 6, Moscow, 117198, Russian Federation; d N. D. Zelinsky Institute of Organic Chemistry RAS, Leninsky Prosp. 47, Moscow, 119991, Russian Federation; e Department of Physics, Faculty of Sciences, Erciyes University, 38039 Kayseri, Turkey; f Department of Physics, Faculty of Science, Eskisehir Technical University, Yunus Emre Campus 26470 Eskisehir, Turkey; g Department of Physics, Faculty of Science, Erciyes University, 38039 Kayseri, Turkey; h Department of Chemistry, M.M.A.M.C (Tribhuvan University) Biratnagar, Nepal

**Keywords:** crystal structure, 1,3-oxazolidine, hydrogen bond, van der Waals inter­actions, Hirshfeld surface analysis

## Abstract

The title compound, C_20_H_24_BrNO_2_, is chiral at the carbon atoms on either side of the oxygen atom of the oxazolidine ring and crystallizes as a racemate. The 1,3-oxazolidine ring adopts an envelope conformation with the N atom in an *endo* position. The mean plane of the oxazolidine ring makes dihedral angles of 77.74 (10) and 45.50 (11)°, respectively, with the 4-bromo­phenol and 1,3,5-tri­methyl­benzene rings. In the crystal, adjacent mol­ecules are connected *via* C—H⋯O hydrogen bonds and C—H⋯π inter­actions into layers parallel to the (200) plane. The packing is strengthened by van der Waals inter­actions between parallel mol­ecular layers. A Hirshfeld surface analysis shows that H⋯H (58.2%), C⋯H/H⋯C (18.9%), and Br⋯H/H⋯Br (11.5%) inter­actions are the most abundant in the crystal packing.

## Abstract 2

In the crystal, neighboring mol­ecules are linked into layers parallel to the (200) plane *via* C—H⋯O hydrogen bonds and C—H⋯π inter­actions. van der Waals inter­actions between parallel mol­ecular layers help to strengthen the packing.

## 1. Chemical context

Functionalization of amine and carbonyl compounds represents a cornerstone of organic synthesis, material science and medicinal chemistry (Zubkov *et al.*, 2018 ▸; Shikhaliyev *et al.*, 2019 ▸; Viswanathan *et al.*, 2019 ▸; Gurbanov *et al.*, 2020 ▸). In particular, the reaction of 1,2-amino alcohols with oxo compounds is an effective tool in the construction of a broad class of organic compounds such as amides, esters, enamino­nes, ureas, carbamates, aziridines, oxazolidines, oxazolines, oxazolidinones, oxazines, pyrroles, pyridones, morpholines, acridinones *etc* (Juhász *et al.*, 2011 ▸; Tamura *et al.*, 2014 ▸; Sepideh *et al.*, 2018 ▸; Khalilov, 2021 ▸). 
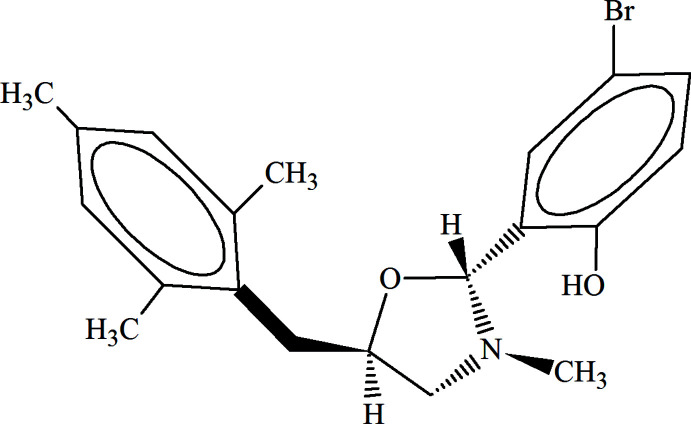


**Figure 1:**
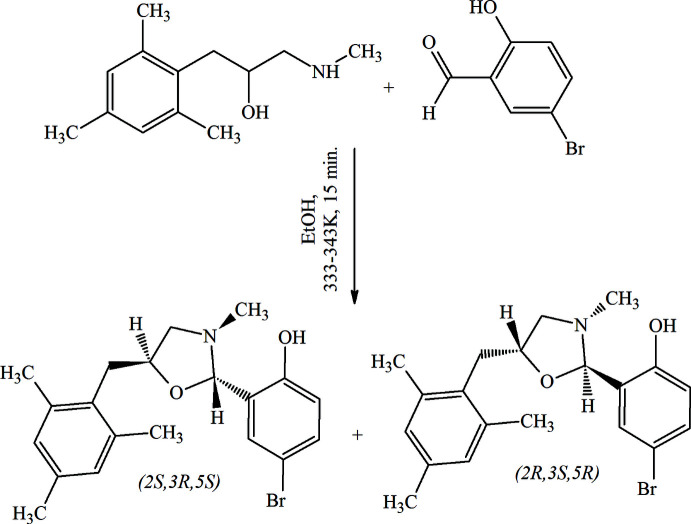
Synthesis of the racemic mixture of 2*R*,3*S*,5*R*- and 2*S*,3*R*,5*S*-oxazolidines.

In the context of our recent studies, herein we report the structural analysis of a 1,3-oxazolidine, synthesized on the base of racemic 1,2-amino alcohol. Theoretically, in the solid state, this 1,3-oxazolidine can exist as eight optical isomers due to two CH and one N-chiral center. However, NMR analysis of the obtained product indicated the formation of a pair of diastereoisomers in a 1:1 ratio (Khalilov, 2021 ▸) and single-crystal X-ray analysis of the racemic mixture confirmed the 2*R*,3*S*,5*R*- and 2*S*,3*R*,5*S*-configuration of these isomers (Fig. 1 ▸).

Thus, in the framework of our ongoing structural studies (Naghiyev *et al.*, 2020 ▸, 2021 ▸, 2022 ▸; Khalilov *et al.*, 2022 ▸), we report the crystal structure and Hirshfeld surface analysis of the racemic title compound, 4-bromo-2-[3-methyl-5-(2,4,6-tri­methyl­benz­yl)oxazolidin-2-yl]phenol.

## 2. Structural commentary

**Figure 2:**
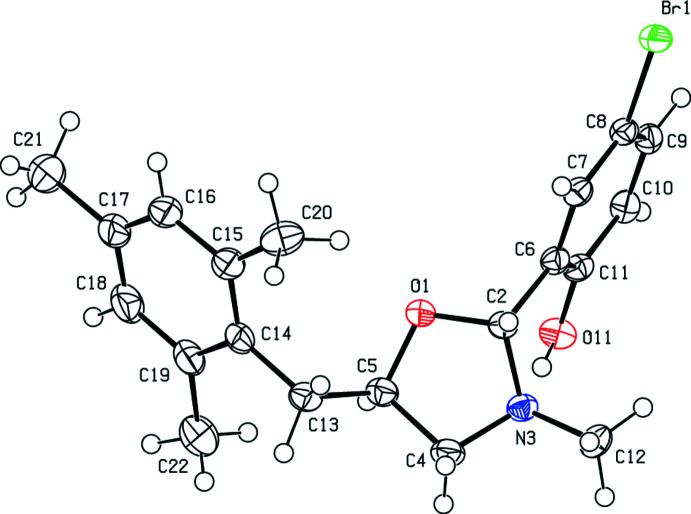
The mol­ecular structure of the title compound. Displacement ellipsoids are drawn at the 50% probability level.

In the title compound, (Fig. 2 ▸), the 1,3-oxazolidine ring (O1/N3/C2/C4/C5) adopts an envelope conformation with the N atom in an *endo* position [the puckering parameters (Cremer & Pople, 1975 ▸) are *Q*(2) = 0.413 (2) Å, φ(2) = 256.7 (3)°]. The mean plane of th oxazolidine ring makes dihedral angles of 77.74 (10) and 45.50 (11)°, respectively, with the 4-bromo­phenol (C6–C11) and the 1,3,5-tri­methyl­benzene (C14–C19) rings. The mol­ecular conformation is stabilized by intra­molecular O11—H11⋯N3 and C20—H20*C*⋯O1 hydrogen bonds (Table 1 ▸). There are two stereogenic centers in the racaemic title compound and the chirality about the C2 and C5 atoms is *R* in the chosen asymmetric unit. The geometric properties of the title compound are normal and consistent with those of related compounds listed in the *Database survey* section.

## 3. Supra­molecular features and Hirshfeld surface analysis

**Figure 3:**
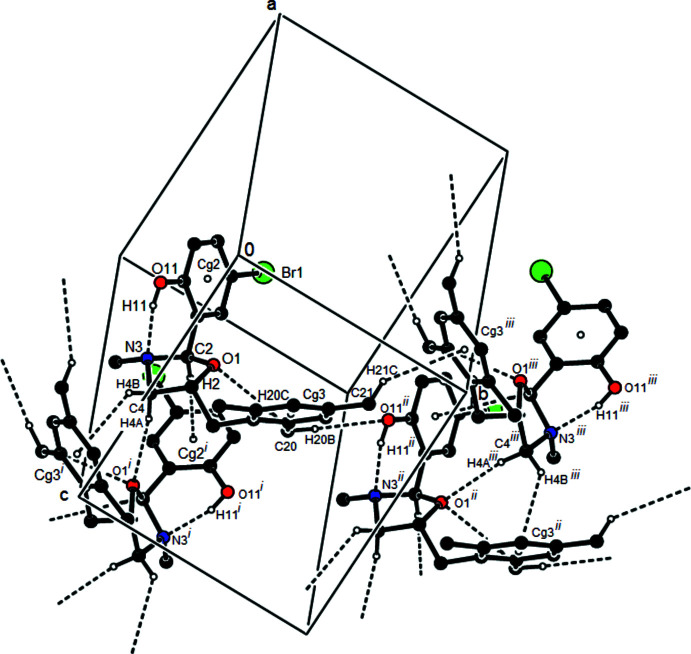
A general view of the C—H⋯O hydrogen bonding and C—H⋯π inter­actions of the title compound. Symmetry codes: (i) *x*, −*y* + 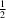 , *z* + 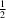 ; (ii) *x*, *y* + 1, *z*; (iii) *x*, −*y* − 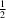 , *z* − 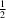 ; (iv) *x*, −*y* + 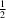 , *z* − 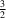 .

In the crystal, adjacent mol­ecules are connected *via* C—H⋯O hydrogen bonds and C—H⋯π inter­actions into layers parallel to the (200) plane (Table 1 ▸; Figs. 3 ▸ and 4 ▸). The packing is strengthened by van der Waals inter­actions between parallel mol­ecular layers.

**Figure 5:**
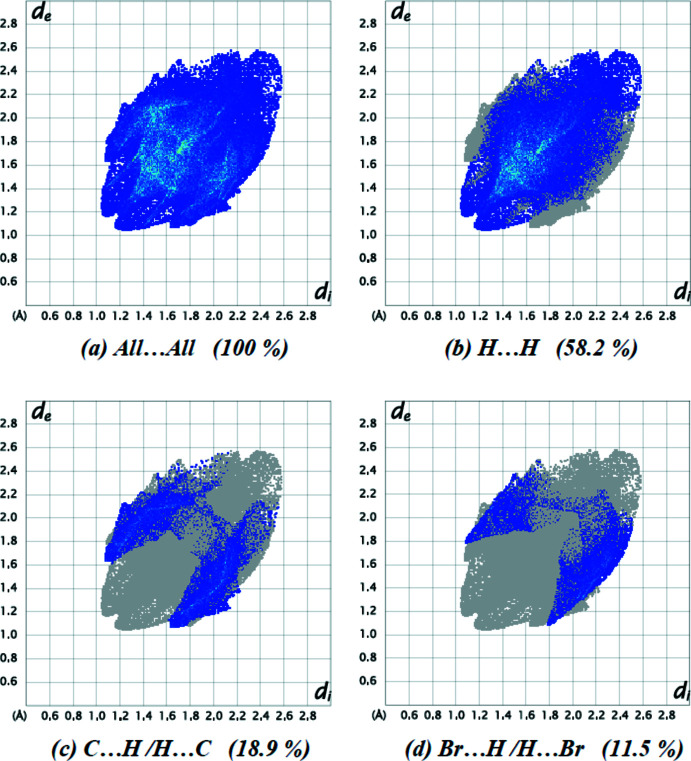
The two-dimensional fingerprint plots of the title compound, showing (*a*) all inter­actions, and delineated into (*b*) H⋯H, (*c*) C⋯H/H⋯C and (*d*) Br⋯H/H⋯Br inter­actions. [*d*_e_ and *d*_i_ represent the distances from a point on the Hirshfeld surface to the nearest atoms outside (external) and inside (inter­nal) the surface, respectively.]

A Hirshfeld surface analysis was performed and the associated two-dimensional fingerprint plots were obtained with *CrystalExplorer17*.5 (Turner *et al.*, 2017 ▸). The overall two-dimensional fingerprint plot for the title compound is given in Fig. 5 ▸ *a*, and those delineated into H⋯H (58.2%), C⋯H/H⋯C (18.9%), and Br⋯H/H⋯Br (11.5%) contacts are shown in Fig. 5 ▸ *b*–*d*, while numerical details of the different contacts are given in Table 2 ▸. The O⋯H/H⋯O (8.3%), C⋯C (1.4%), Br⋯C/C⋯Br (1.0%), Br⋯O/O⋯Br (0.5%) and Br⋯Br (0.3%) contacts have little directional influence on the mol­ecular packing. A a result, in the crystal packing, C—H⋯π (ring) and van der Waals inter­actions are dominant.

## 4. Database survey

A search of the Cambridge Structural Database (CSD, Version 5.42, update of September 2021; Groom *et al.*, 2016 ▸) for similar structures with a 1,3-oxazolidine ring showed that the five most closely related to the title compound are (*S*)-5-chloro-*N*-({2-oxo-3-[4-(3-oxomorpholin-4-yl)phen­yl]oxa­zolidin-5-yl}meth­yl)-thio­phene-2-carboxamide [(I): Shen *et al.*, 2018 ▸], 2,2-di­chloro-1-(2-phenyl-1,3-oxazolidin-3-yl)ethan­one [(II): Ye *et al.*, 2010 ▸], (4-benzyl-2-oxo-1,3-oxazolidin-5-yl)- methyl methane­sulfonate [(III): Cunico *et al.*, 2010 ▸], 2-bromo-4-(3,4-dimethyl-5-phenyl-1,3-oxazolidin-2-yl)-6-meth­oxy­phenol [(IV): Hariono *et al.*, 2012 ▸] and (*R*)-2-phen­oxy-1-(4-phenyl-2-sulfanyl­idene-1,3-oxazolidin-3-yl)ethanone [(V): Caracelli *et al.*, 2011 ▸].

In the crystal of (I), classical N—H⋯O hydrogen bonds and weak C— H⋯O hydrogen bonds link the mol­ecules into a three-dimensional supra­molecular architecture. In (II), mol­ecules are linked by weak inter­molecular C—H⋯O hydrogen bonds, forming one-dimensional chains. In the crystal of (III), N—H⋯O hydrogen bonds, involving one of the sulfur-bound oxo groups as acceptor, lead to the formation of supra­molecular chains along the *b*-axis direction. These chains are reinforced by C—H⋯O contacts, with the carbonyl O atom accepting three such inter­actions. In (IV), adjacent mol­ecules are connected *via* O—H⋯O and C—H⋯O hydrogen bonds and C—H⋯π inter­actions into a zigzag chain along the *b*-axis direction. In (V), mol­ecules are linked into supra­molecular arrays two mol­ecules thick in the *bc* plane through C—H⋯O, C—H⋯S and C—H⋯π inter­actions.

## 5. Synthesis and crystallization

The title compound was synthesized using our recently reported procedure (Khalilov, 2021 ▸), and colorless needle-like crystals were obtained upon recrystallization from an ethanol/water solution.

## 6. Refinement

Crystal data, data collection and structure refinement details are summarized in Table 3 ▸. All C-bound H atoms were placed at calculated positions and refined using a riding model, with C—H = 0.95 to 1.00 Å, and with *U*_iso_(H) = 1.2 or 1.5*U*_eq_(C). The hydroxyl H atom was found in a difference-Fourier map and was refined freely.

## Supplementary Material

Crystal structure: contains datablock(s) I. DOI: 10.1107/S2056989022005928/tx2051sup1.cif

Structure factors: contains datablock(s) I. DOI: 10.1107/S2056989022005928/tx2051Isup2.hkl

Supporting information file. DOI: 10.1107/S2056989022005928/tx2051Isup3.cml

CCDC reference: 2176709

Additional supporting information:  crystallographic information; 3D view; checkCIF report

## Figures and Tables

**Figure 4:**
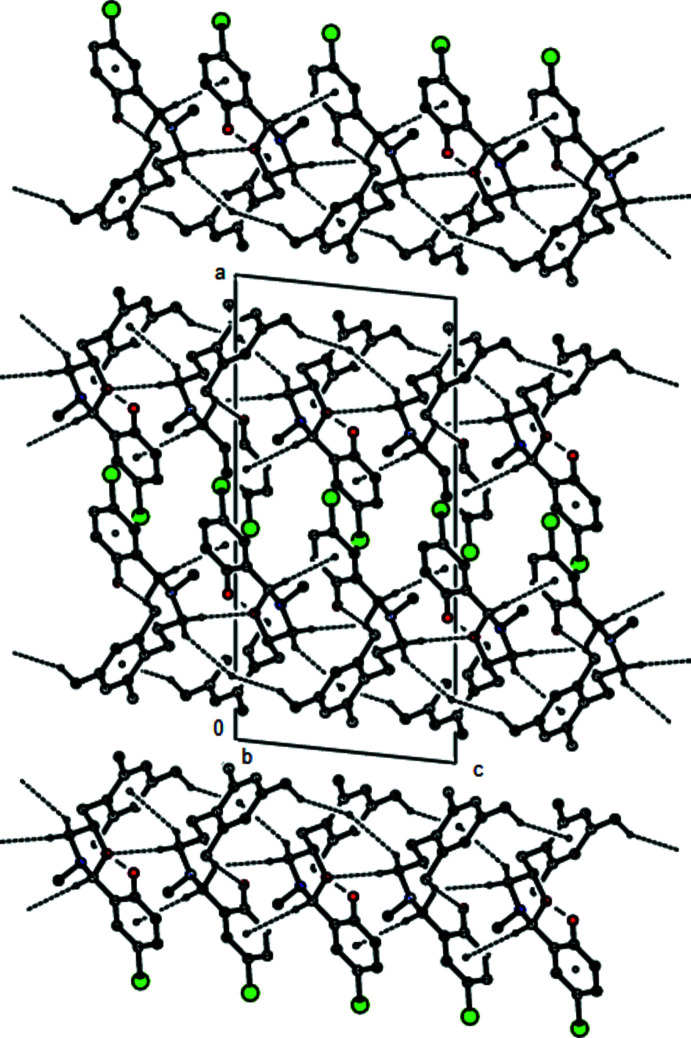
Packing view of the title compound along the *b* axis with the inter­actions depicted as in Fig. 3 ▸.

**Table 1:** Hydrogen-bond geometry (Å, °) *Cg*2 and *Cg*3 are the centroids of the 4-bromo­phenol (C6–C11) and 1,3,5-tri­methyl­benzene (C14–C19) rings, respectively.

*D*—H⋯*A*	*D*—H	H⋯*A*	*D*⋯*A*	*D*—H⋯*A*
O11—H11⋯N3	0.81 (4)	1.89 (4)	2.644 (2)	155 (3)
C4—H4*A*⋯O1^i^	0.99	2.58	3.564 (2)	171
C20—H20*B*⋯O11^ii^	0.98	2.57	3.548 (3)	173
C20—H20*C*⋯O1	0.98	2.55	3.332 (3)	136
C2—H2⋯*Cg*2^i^	1.00	2.91	3.908 (2)	176
C4—H4*B*⋯*Cg*3^i^	0.99	2.88	3.622 (2)	132
C21—H21*C*⋯*Cg*3^iii^	0.98	2.93	3.723 (4)	138

Symmetry codes: (i) 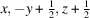 ; (ii) 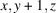 ; (iii) 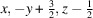 .

**Table 2:** Summary of short inter­atomic contacts (Å) in the title compound

Contact	Distance	Symmetry operation
Br1⋯H10	2.96	1 − *x*, 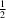 + *y*, 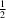 − *z*
Br1⋯C12	3.598	1 − *x*, 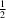 + *y*, 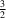 − *z*
C9⋯C8	3.409	1 − *x*, −*y*, 1 − *z*
H9⋯H7	2.45	*x*, 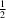 − *y*, − 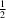 + *z*
H11⋯H20*B*	2.35	*x*, −1 + *y*, *z*
C15⋯H21*C*	2.80	*x*, 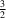 − *y*, 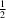 + *z*
H22*B*⋯C18	3.07	−*x*, 1 − *y*, 1 − *z*
H21*B*⋯H22*B*	2.51	−*x*, 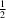 + *y*, 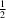 − *z*

**Table 3:** Experimental details

Crystal data	
Chemical formula	C_20_H_24_BrNO_2_
*M*_r_	390.30
Crystal system, space group	Monoclinic, *P*2_1_/*c*
Temperature (K)	100
*a*, *b*, *c* (Å)	21.1019 (3), 9.01359 (11), 10.03985 (11)
β (°)	96.1425 (11)
*V* (Å^3^)	1898.66 (4)
*Z*	4
Radiation type	Cu *K*α
μ (mm^−1^)	3.03
Crystal size (mm)	0.32 × 0.04 × 0.03

Data collection	
Diffractometer	XtaLAB Synergy, Dualflex, HyPix
Absorption correction	Multi-scan (*CrysAlis PRO*; Rigaku OD, 2021 ▸)
*T*_min_, *T*_max_	0.424, 0.882
No. of measured, independent and observed [*I* > 2σ(*I*)] reflections	21431, 4096, 3783
*R*_int_	0.043
(sin θ/λ)_max_ (Å^−1^)	0.638

Refinement	
*R*[*F* ^2^ > 2σ(*F* ^2^)], *wR*(*F* ^2^), *S*	0.033, 0.096, 1.07
No. of reflections	4096
No. of parameters	225
H-atom treatment	H atoms treated by a mixture of independent and constrained refinement
Δρ_max_, Δρ_min_ (e Å^−3^)	0.58, −0.60

Computer programs: *CrysAlis PRO* (Rigaku OD, 2021 ▸), *SHELXT* (Sheldrick, 2015*a* ▸), *SHELXL2018* (Sheldrick, 2015*b* ▸), *ORTEP-3 for Windows* (Farrugia, 2012 ▸) and *PLATON* (Spek, 2020 ▸).

## supplementary crystallographic information

### Crystal data

**Table:**

C_20_H_24_BrNO_2_	*F*(000) = 808
*M**_r_* = 390.30	*D*_x_ = 1.365 Mg m^−^^3^
Monoclinic, *P*2_1_/*c*	Cu *K*α radiation, λ = 1.54184 Å
*a* = 21.1019 (3) Å	Cell parameters from 13703 reflections
*b* = 9.01359 (11) Å	θ = 2.1–78.6°
*c* = 10.03985 (11) Å	µ = 3.03 mm^−^^1^
β = 96.1425 (11)°	*T* = 100 K
*V* = 1898.66 (4) Å^3^	Needle, colourless
*Z* = 4	0.32 × 0.04 × 0.03 mm

### Data collection

**Table:**

XtaLAB Synergy, Dualflex, HyPix diffractometer	3783 reflections with *I* > 2σ(*I*)
Radiation source: micro-focus sealed X-ray tube	*R*_int_ = 0.043
φ and ω scans	θ_max_ = 79.6°, θ_min_ = 2.1°
Absorption correction: multi-scan (CrysAlisPro; Rigaku OD, 2021)	*h* = −25→26
*T*_min_ = 0.424, *T*_max_ = 0.882	*k* = −11→11
21431 measured reflections	*l* = −12→10
4096 independent reflections	

### Refinement

**Table:**

Refinement on *F*^2^	Primary atom site location: difference Fourier map
Least-squares matrix: full	Secondary atom site location: difference Fourier map
*R*[*F*^2^ > 2σ(*F*^2^)] = 0.033	Hydrogen site location: mixed
*wR*(*F*^2^) = 0.096	H atoms treated by a mixture of independent and constrained refinement
*S* = 1.07	*w* = 1/[σ^2^(*F*_o_^2^) + (0.055*P*)^2^ + 1.11*P*] where *P* = (*F*_o_^2^ + 2*F*_c_^2^)/3
4096 reflections	(Δ/σ)_max_ = 0.001
225 parameters	Δρ_max_ = 0.58 e Å^−^^3^
0 restraints	Δρ_min_ = −0.60 e Å^−^^3^

### Special details

**Table:**

Experimental. CrysAlisPro 1.171.41.117a (Rigaku OD, 2021) Empirical absorption correction using spherical harmonics, implemented in SCALE3 ABSPACK scaling algorithm.
Geometry. All esds (except the esd in the dihedral angle between two l.s. planes) are estimated using the full covariance matrix. The cell esds are taken into account individually in the estimation of esds in distances, angles and torsion angles; correlations between esds in cell parameters are only used when they are defined by crystal symmetry. An approximate (isotropic) treatment of cell esds is used for estimating esds involving l.s. planes.

### Fractional atomic coordinates and isotropic or equivalent isotropic displacement parameters (Å^2^)

**Table:**

	*x*	*y*	*z*	*U*_iso_*/*U*_eq_	
Br1	0.54220 (2)	0.31515 (2)	0.43375 (2)	0.02813 (9)	
O1	0.27633 (7)	0.25150 (17)	0.58702 (14)	0.0298 (3)	
C2	0.32957 (9)	0.1726 (2)	0.65118 (19)	0.0232 (4)	
H2	0.3517	0.2334	0.7256	0.028*	
N3	0.30119 (8)	0.03941 (18)	0.70533 (15)	0.0240 (3)	
C4	0.24396 (10)	0.0998 (2)	0.7578 (2)	0.0286 (4)	
H4A	0.2548	0.1518	0.8441	0.034*	
H4B	0.2124	0.0210	0.7696	0.034*	
C5	0.21970 (10)	0.2075 (2)	0.6467 (2)	0.0275 (4)	
H5	0.1901	0.1541	0.5785	0.033*	
C6	0.37483 (9)	0.1367 (2)	0.54907 (18)	0.0226 (4)	
C7	0.42805 (9)	0.2251 (2)	0.53991 (18)	0.0235 (4)	
H7	0.4364	0.3066	0.5991	0.028*	
C8	0.46904 (9)	0.1942 (2)	0.44404 (19)	0.0229 (4)	
C9	0.45780 (9)	0.0753 (2)	0.35676 (18)	0.0238 (4)	
H9	0.4865	0.0541	0.2925	0.029*	
C10	0.40439 (10)	−0.0118 (2)	0.36452 (18)	0.0255 (4)	
H10	0.3961	−0.0925	0.3043	0.031*	
C11	0.36252 (9)	0.0175 (2)	0.45995 (18)	0.0237 (4)	
O11	0.31123 (7)	−0.07205 (17)	0.46493 (15)	0.0287 (3)	
H11	0.2980 (17)	−0.053 (4)	0.536 (4)	0.050 (9)*	
C12	0.34457 (10)	−0.0365 (2)	0.8069 (2)	0.0295 (4)	
H12A	0.3823	−0.0707	0.7665	0.044*	
H12B	0.3228	−0.1218	0.8418	0.044*	
H12C	0.3577	0.0323	0.8803	0.044*	
C13	0.18595 (10)	0.3429 (2)	0.6965 (2)	0.0293 (4)	
H13A	0.1502	0.3093	0.7453	0.035*	
H13B	0.2162	0.3982	0.7606	0.035*	
C14	0.16029 (10)	0.4466 (2)	0.5851 (2)	0.0288 (4)	
C15	0.19383 (11)	0.5761 (2)	0.5574 (2)	0.0301 (4)	
C16	0.16792 (12)	0.6723 (3)	0.4563 (2)	0.0361 (5)	
H16	0.1904	0.7603	0.4389	0.043*	
C17	0.11055 (12)	0.6426 (3)	0.3812 (3)	0.0430 (6)	
C18	0.07858 (11)	0.5135 (4)	0.4079 (3)	0.0452 (6)	
H18	0.0393	0.4915	0.3564	0.054*	
C19	0.10236 (10)	0.4145 (3)	0.5084 (2)	0.0365 (5)	
C20	0.25709 (13)	0.6141 (3)	0.6335 (2)	0.0393 (5)	
H20A	0.2512	0.6344	0.7273	0.059*	
H20B	0.2748	0.7021	0.5938	0.059*	
H20C	0.2865	0.5304	0.6291	0.059*	
C21	0.08362 (15)	0.7492 (5)	0.2732 (3)	0.0650 (10)	
H21A	0.0776	0.8468	0.3131	0.097*	
H21B	0.0425	0.7119	0.2318	0.097*	
H21C	0.1133	0.7581	0.2050	0.097*	
C22	0.06445 (12)	0.2765 (4)	0.5315 (3)	0.0523 (7)	
H22A	0.0859	0.1897	0.4985	0.079*	
H22B	0.0217	0.2853	0.4835	0.079*	
H22C	0.0611	0.2651	0.6276	0.079*	

### Atomic displacement parameters (Å^2^)

**Table:**

	*U*^11^	*U*^22^	*U*^33^	*U*^12^	*U*^13^	*U*^23^
Br1	0.02612 (13)	0.02966 (14)	0.02922 (14)	−0.00322 (7)	0.00584 (9)	0.00477 (7)
O1	0.0269 (7)	0.0344 (8)	0.0296 (7)	0.0054 (6)	0.0103 (6)	0.0108 (6)
C2	0.0242 (9)	0.0241 (9)	0.0216 (8)	−0.0010 (7)	0.0035 (7)	0.0011 (7)
N3	0.0258 (8)	0.0261 (8)	0.0201 (7)	−0.0015 (6)	0.0024 (6)	0.0029 (6)
C4	0.0291 (9)	0.0332 (10)	0.0245 (9)	−0.0019 (8)	0.0076 (7)	0.0037 (8)
C5	0.0270 (10)	0.0315 (10)	0.0249 (9)	−0.0003 (8)	0.0068 (7)	0.0021 (8)
C6	0.0254 (9)	0.0250 (9)	0.0173 (8)	0.0018 (7)	0.0018 (6)	0.0021 (7)
C7	0.0269 (9)	0.0231 (8)	0.0201 (8)	0.0005 (7)	0.0007 (7)	0.0015 (7)
C8	0.0223 (9)	0.0250 (9)	0.0212 (9)	−0.0002 (7)	0.0014 (7)	0.0040 (7)
C9	0.0257 (9)	0.0273 (9)	0.0183 (8)	0.0048 (7)	0.0018 (6)	0.0014 (7)
C10	0.0296 (9)	0.0266 (9)	0.0197 (8)	0.0039 (8)	0.0005 (7)	−0.0025 (7)
C11	0.0260 (9)	0.0238 (9)	0.0209 (8)	−0.0015 (7)	0.0002 (7)	0.0039 (7)
O11	0.0297 (7)	0.0317 (8)	0.0249 (7)	−0.0075 (6)	0.0040 (6)	−0.0043 (6)
C12	0.0347 (10)	0.0290 (10)	0.0239 (9)	0.0027 (8)	−0.0009 (8)	0.0043 (8)
C13	0.0312 (10)	0.0322 (10)	0.0260 (10)	0.0005 (8)	0.0093 (8)	0.0018 (8)
C14	0.0282 (9)	0.0335 (10)	0.0264 (9)	0.0076 (8)	0.0109 (7)	0.0004 (8)
C15	0.0369 (11)	0.0319 (10)	0.0231 (9)	0.0056 (8)	0.0102 (8)	−0.0007 (8)
C16	0.0421 (13)	0.0376 (12)	0.0314 (11)	0.0092 (9)	0.0167 (10)	0.0062 (9)
C17	0.0364 (12)	0.0587 (15)	0.0361 (12)	0.0159 (11)	0.0142 (10)	0.0169 (11)
C18	0.0252 (10)	0.0668 (17)	0.0435 (13)	0.0095 (11)	0.0032 (9)	0.0111 (12)
C19	0.0240 (9)	0.0471 (13)	0.0392 (11)	0.0059 (9)	0.0072 (8)	0.0041 (10)
C20	0.0515 (14)	0.0358 (12)	0.0306 (11)	−0.0086 (10)	0.0039 (10)	−0.0001 (9)
C21	0.0446 (15)	0.094 (3)	0.0574 (17)	0.0211 (17)	0.0119 (13)	0.0422 (19)
C22	0.0265 (11)	0.0609 (17)	0.0689 (18)	−0.0046 (12)	0.0016 (11)	0.0095 (15)

### Geometric parameters (Å, º)

**Table:**

Br1—C8	1.9019 (19)	C12—H12B	0.9800
O1—C2	1.424 (2)	C12—H12C	0.9800
O1—C5	1.448 (2)	C13—C14	1.513 (3)
C2—N3	1.472 (2)	C13—H13A	0.9900
C2—C6	1.509 (3)	C13—H13B	0.9900
C2—H2	1.0000	C14—C19	1.404 (3)
N3—C12	1.465 (2)	C14—C15	1.407 (3)
N3—C4	1.472 (3)	C15—C16	1.401 (3)
C4—C5	1.525 (3)	C15—C20	1.505 (3)
C4—H4A	0.9900	C16—C17	1.382 (4)
C4—H4B	0.9900	C16—H16	0.9500
C5—C13	1.524 (3)	C17—C18	1.385 (4)
C5—H5	1.0000	C17—C21	1.513 (4)
C6—C7	1.388 (3)	C18—C19	1.399 (4)
C6—C11	1.404 (3)	C18—H18	0.9500
C7—C8	1.389 (3)	C19—C22	1.510 (4)
C7—H7	0.9500	C20—H20A	0.9800
C8—C9	1.388 (3)	C20—H20B	0.9800
C9—C10	1.383 (3)	C20—H20C	0.9800
C9—H9	0.9500	C21—H21A	0.9800
C10—C11	1.396 (3)	C21—H21B	0.9800
C10—H10	0.9500	C21—H21C	0.9800
C11—O11	1.356 (2)	C22—H22A	0.9800
O11—H11	0.81 (4)	C22—H22B	0.9800
C12—H12A	0.9800	C22—H22C	0.9800
			
C2—O1—C5	108.79 (14)	H12A—C12—H12C	109.5
O1—C2—N3	104.01 (15)	H12B—C12—H12C	109.5
O1—C2—C6	109.02 (15)	C14—C13—C5	113.25 (17)
N3—C2—C6	112.83 (16)	C14—C13—H13A	108.9
O1—C2—H2	110.3	C5—C13—H13A	108.9
N3—C2—H2	110.3	C14—C13—H13B	108.9
C6—C2—H2	110.3	C5—C13—H13B	108.9
C12—N3—C2	112.92 (16)	H13A—C13—H13B	107.7
C12—N3—C4	113.51 (15)	C19—C14—C15	119.3 (2)
C2—N3—C4	102.26 (15)	C19—C14—C13	120.0 (2)
N3—C4—C5	101.41 (15)	C15—C14—C13	120.7 (2)
N3—C4—H4A	111.5	C16—C15—C14	119.4 (2)
C5—C4—H4A	111.5	C16—C15—C20	118.9 (2)
N3—C4—H4B	111.5	C14—C15—C20	121.7 (2)
C5—C4—H4B	111.5	C17—C16—C15	121.8 (2)
H4A—C4—H4B	109.3	C17—C16—H16	119.1
O1—C5—C13	110.61 (17)	C15—C16—H16	119.1
O1—C5—C4	104.45 (16)	C16—C17—C18	118.2 (2)
C13—C5—C4	113.71 (17)	C16—C17—C21	120.5 (3)
O1—C5—H5	109.3	C18—C17—C21	121.3 (3)
C13—C5—H5	109.3	C17—C18—C19	122.1 (2)
C4—C5—H5	109.3	C17—C18—H18	119.0
C7—C6—C11	119.50 (17)	C19—C18—H18	119.0
C7—C6—C2	119.82 (17)	C18—C19—C14	119.2 (2)
C11—C6—C2	120.64 (17)	C18—C19—C22	118.8 (2)
C6—C7—C8	119.94 (18)	C14—C19—C22	122.0 (2)
C6—C7—H7	120.0	C15—C20—H20A	109.5
C8—C7—H7	120.0	C15—C20—H20B	109.5
C9—C8—C7	121.00 (18)	H20A—C20—H20B	109.5
C9—C8—Br1	119.54 (14)	C15—C20—H20C	109.5
C7—C8—Br1	119.46 (15)	H20A—C20—H20C	109.5
C10—C9—C8	119.20 (17)	H20B—C20—H20C	109.5
C10—C9—H9	120.4	C17—C21—H21A	109.5
C8—C9—H9	120.4	C17—C21—H21B	109.5
C9—C10—C11	120.70 (18)	H21A—C21—H21B	109.5
C9—C10—H10	119.7	C17—C21—H21C	109.5
C11—C10—H10	119.7	H21A—C21—H21C	109.5
O11—C11—C10	118.61 (18)	H21B—C21—H21C	109.5
O11—C11—C6	121.74 (17)	C19—C22—H22A	109.5
C10—C11—C6	119.65 (18)	C19—C22—H22B	109.5
C11—O11—H11	105 (2)	H22A—C22—H22B	109.5
N3—C12—H12A	109.5	C19—C22—H22C	109.5
N3—C12—H12B	109.5	H22A—C22—H22C	109.5
H12A—C12—H12B	109.5	H22B—C22—H22C	109.5
N3—C12—H12C	109.5		
			
C5—O1—C2—N3	23.8 (2)	C7—C6—C11—O11	179.93 (17)
C5—O1—C2—C6	144.39 (16)	C2—C6—C11—O11	−2.2 (3)
O1—C2—N3—C12	−163.70 (15)	C7—C6—C11—C10	0.9 (3)
C6—C2—N3—C12	78.3 (2)	C2—C6—C11—C10	178.71 (17)
O1—C2—N3—C4	−41.36 (18)	O1—C5—C13—C14	64.6 (2)
C6—C2—N3—C4	−159.35 (16)	C4—C5—C13—C14	−178.21 (18)
C12—N3—C4—C5	163.78 (17)	C5—C13—C14—C19	80.4 (2)
C2—N3—C4—C5	41.84 (18)	C5—C13—C14—C15	−99.8 (2)
C2—O1—C5—C13	125.28 (18)	C19—C14—C15—C16	1.7 (3)
C2—O1—C5—C4	2.6 (2)	C13—C14—C15—C16	−178.05 (18)
N3—C4—C5—O1	−27.6 (2)	C19—C14—C15—C20	−178.2 (2)
N3—C4—C5—C13	−148.32 (17)	C13—C14—C15—C20	2.1 (3)
O1—C2—C6—C7	98.9 (2)	C14—C15—C16—C17	−1.0 (3)
N3—C2—C6—C7	−146.13 (17)	C20—C15—C16—C17	178.9 (2)
O1—C2—C6—C11	−79.0 (2)	C15—C16—C17—C18	−0.1 (4)
N3—C2—C6—C11	36.0 (2)	C15—C16—C17—C21	179.6 (2)
C11—C6—C7—C8	−0.7 (3)	C16—C17—C18—C19	0.5 (4)
C2—C6—C7—C8	−178.59 (17)	C21—C17—C18—C19	−179.2 (3)
C6—C7—C8—C9	−0.2 (3)	C17—C18—C19—C14	0.2 (4)
C6—C7—C8—Br1	−179.44 (14)	C17—C18—C19—C22	179.8 (3)
C7—C8—C9—C10	1.0 (3)	C15—C14—C19—C18	−1.3 (3)
Br1—C8—C9—C10	−179.76 (14)	C13—C14—C19—C18	178.4 (2)
C8—C9—C10—C11	−0.9 (3)	C15—C14—C19—C22	179.1 (2)
C9—C10—C11—O11	−179.15 (17)	C13—C14—C19—C22	−1.1 (3)
C9—C10—C11—C6	−0.1 (3)		

### Hydrogen-bond geometry (Å, º)

*Cg*2 and *Cg*3 are the centroids of the 4-bromophenol (C6–C11) and 1,3,5-trimethylbenzene (C14–C19) rings, respectively.

**Table:**

*D*—H···*A*	*D*—H	H···*A*	*D*···*A*	*D*—H···*A*
O11—H11···N3	0.81 (4)	1.89 (4)	2.644 (2)	155 (3)
C4—H4*A*···O1^i^	0.99	2.58	3.564 (2)	171
C20—H20*B*···O11^ii^	0.98	2.57	3.548 (3)	173
C20—H20*C*···O1	0.98	2.55	3.332 (3)	136
C2—H2···*Cg*2^i^	1.00	2.91	3.908 (2)	176
C4—H4*B*···*Cg*3^i^	0.99	2.88	3.622 (2)	132
C21—H21*C*···*Cg*3^iii^	0.98	2.93	3.723 (4)	138

Symmetry codes: (i) *x*, −*y*+1/2, *z*+1/2; (ii) *x*, *y*+1, *z*; (iii) *x*, −*y*+3/2, *z*−1/2.

## References

[bb1] Caracelli, I., Coelho, D. C. S., Olivato, P. R., Correra, T. C., Rodrigues, A. & Tiekink, E. R. T. (2011). *Acta Cryst.* E**67**, o2755–o2756. 10.1107/S160053681103858X PMC3201535 22065310

[bb2] Cremer, D. & Pople, J. A. (1975). *J. Am. Chem. Soc.* **97**, 1354–1358.

[bb3] Cunico, W., Gomes, C. R. B., Tiekink, E. R. T., Vellasco Junior, W. T., Wardell, J. L. & Wardell, S. M. S. V. (2010). *Acta Cryst.* E**66**, o267–o268. 10.1107/S1600536809055020 PMC2979937 21579707

[bb4] Farrugia, L. J. (2012). *J. Appl. Cryst.* **45**, 849–854.

[bb5] Groom, C. R., Bruno, I. J., Lightfoot, M. P. & Ward, S. C. (2016). *Acta Cryst.* B**72**, 171–179. 10.1107/S2052520616003954 PMC4822653 27048719

[bb6] Gurbanov, A. V., Kuznetsov, M. L., Demukhamedova, S. D., Alieva, I. N., Godjaev, N. M., Zubkov, F. I., Mahmudov, K. T. & Pombeiro, A. J. L. (2020). *CrystEngComm*, **22**, 628–633.

[bb7] Hariono, M., Ngah, N., Wahab, H. A. & Abdul Rahim, A. S. (2012). *Acta Cryst.* E**68**, o35–o36. 10.1107/S1600536811051269 PMC3254397 22259539

[bb8] Juhász, M., Lázár, L. & Fülöp, F. (2011). *Tetrahedron Asymmetry*, **22**, 2012–2017.

[bb9] Khalilov, A. N. (2021). *Rev. Roum. Chim.* **66**, 719–723.

[bb10] Khalilov, A. N., Khrustalev, V. N., Tereshina, T. A., Akkurt, M., Rzayev, R. M., Akobirshoeva, A. A. & Mamedov, İ. G. (2022). *Acta Cryst.* E**78**, 525–529. 10.1107/S2056989022004297 PMC9069515 35547793

[bb11] Naghiyev, F. N., Akkurt, M., Askerov, R. K., Mamedov, I. G., Rzayev, R. M., Chyrka, T. & Maharramov, A. M. (2020). *Acta Cryst.* E**76**, 720–723. 10.1107/S2056989020005381 PMC7199244 32431939

[bb12] Naghiyev, F. N., Khrustalev, V. N., Novikov, A. P., Akkurt, M., Rzayev, R. M., Akobirshoeva, A. A. & Mamedov, I. G. (2022). *Acta Cryst.* E**78**, 554–558. 10.1107/S2056989022004741 PMC9431780 36072149

[bb13] Naghiyev, F. N., Tereshina, T. A., Khrustalev, V. N., Akkurt, M., Rzayev, R. M., Akobirshoeva, A. A. & Mamedov, İ. G. (2021). *Acta Cryst.* E**77**, 516–521. 10.1107/S2056989021003583 PMC8100256 34026256

[bb14] Rigaku OD (2021). *CrysAlis PRO*. Rigaku Oxford Diffraction, Yarnton, England.

[bb15] Sepideh, F., Zare, F. L., Mohammad, N., Robab, M. & Esmail, V. (2018). *J. CO2 Util.*, **25**, 194-204.

[bb16] Sheldrick, G. M. (2015*a*). *Acta Cryst.* A**71**, 3–8.

[bb17] Sheldrick, G. M. (2015*b*). *Acta Cryst.* C**71**, 3–8.

[bb18] Shen, J., Tang, G.-P. & Hu, X.-R. (2018). *Acta Cryst.* E**74**, 51–54. 10.1107/S2056989017017819 PMC5778484 29416890

[bb19] Shikhaliyev, N. Q., Kuznetsov, M. L., Maharramov, A. M., Gurbanov, A. V., Ahmadova, N. E., Nenajdenko, V. G., Mahmudov, K. T. & Pombeiro, A. J. L. (2019). *CrystEngComm*, **21**, 5032–5038.

[bb20] Spek, A. L. (2020). *Acta Cryst.* E**76**, 1–11. 10.1107/S2056989019016244 PMC6944088 31921444

[bb21] Tamura, M., Honda, M., Nakagawa, Y. & Tomishige, K. (2014). *J. Chem. Technol. Biotechnol.* **89**, 19–33.

[bb22] Turner, M. J., McKinnon, J. J., Wolff, S. K., Grimwood, D. J., Spackman, M. A., Jayatilaka, D. & Spackman, M. A. (2017). *Crystal Explorer17*. University of Western Australia.

[bb23] Viswanathan, A., Kute, D., Musa, A., Konda Mani, S., Sipilä, V., Emmert-Streib, F., Zubkov, F. I., Gurbanov, A. V., Yli-Harja, O. & Kandhavelu, M. (2019). *Eur. J. Med. Chem.* **166**, 291–303. 10.1016/j.ejmech.2019.01.021 30731398

[bb24] Ye, F., Fu, Y. & Zhao, S. (2010). *Acta Cryst.* E**66**, o445. 10.1107/S1600536810002461 PMC2979863 21579860

[bb25] Zubkov, F. I., Mertsalov, D. F., Zaytsev, V. P., Varlamov, A. V., Gurbanov, A. V., Dorovatovskii, P. V., Timofeeva, T. V., Khrustalev, V. N. & Mahmudov, K. T. (2018). *J. Mol. Liq.* **249**, 949–952.

